# Financial Strain Over the Life Course and Health Among Older Adults: A Rural-Urban Comparison

**DOI:** 10.1093/geroni/igaf122.2458

**Published:** 2025-12-31

**Authors:** Rong Fu

**Affiliations:** Siena University, Loudonville, New York, United States

## Abstract

As economic hardship and insecurity increase globally, understanding how subjective socioeconomic status influences health beyond objective measures is essential. This study examines the impact of childhood and adulthood financial strain on health among older adults in mainland China, with attention to potential rural-urban differences. Data were derived from two waves of the Chinese Longitudinal Healthy Longevity Survey (CLHLS). The final sample included 8,175 adults aged 62 and older. Statistical analyses were conducted separately for rural and urban populations. Multiple imputation techniques were used to handle missing data. The findings indicate that both childhood and current financial strain were significantly associated with poor health among urban older adults, even after adjusting for other risk factors such as household income and poverty. In contrast, among rural older adults, only current perceived financial strain was significantly linked to poor health. This study highlights the need for policies that address the specific challenges of both urban and rural residents. Expanding healthcare access for low-income populations and introducing early interventions to reduce the long-term effects of childhood poverty could make a difference.

